# Impact of Mosquito Age and Insecticide Exposure on Susceptibility of *Aedes albopictus* (Diptera: Culicidae) to Infection with Zika Virus

**DOI:** 10.3390/pathogens7030067

**Published:** 2018-08-12

**Authors:** Heidi Knecht, Stephanie L. Richards, Jo Anne G. Balanay, Avian V. White

**Affiliations:** Environmental Health Sciences Program, Department of Health Education and Promotion, College of Health and Human Performance, East Carolina University, Greenville, NC 27858, USA knechth16@students.ecu.edu (H.K.); balanayj@ecu.edu (J.A.G.B.); whiteav15@ecu.edu (A.V.W.)

**Keywords:** vector competence, mosquito age, Zika, insecticides

## Abstract

Zika virus (ZIKV) is primarily transmitted to humans by *Aedes aegypti* and *Ae. albopictus*. Vector–virus interactions influencing vector competence vary and depend on biological and environmental factors. A mosquito’s chronological age may impact its immune response against virus infection. Insecticides, source reduction, and/or public education are currently the best defense against mosquitoes that transmit ZIKV. This study assessed the effects of a mosquito’s chronological age at time of infection on its response to ZIKV infection. We exposed young (6–7 d post-emergence) and old (11–12 d post-emergence) *Ae. albopictus* to a sublethal dose of bifenthrin prior to oral exposure to blood meals containing ZIKV (7-day incubation period). Old mosquitoes experienced a significantly (*p* < 0.01) higher rate of mortality than young mosquitoes. Significantly higher ZIKV body titers (*p* < 0.01) were observed in the old control group compared to the young control group. Significantly higher (*p* < 0.01) ZIKV dissemination rates and leg titers (*p* < 0.01) were observed in old bifenthrin-exposed mosquitoes compared to old control mosquitoes or young bifenthrin-exposed or control mosquitoes. Hence, bifenthrin exposure may increase the potential for virus transmission; however, the degree of these impacts varies with mosquito age. Impacts of insecticides should be considered in risk assessments of potential vector populations.

## 1. Introduction

In the United States there has been widespread concern about a potential epidemic caused by Zika virus (ZIKV; family Flaviviridae, genus Flavivirus). This arbovirus is primarily transmitted to humans by *Aedes aegypti* (Linneaus, 1762), and *Ae. albopictus* (Skuse, 1895); however, it can also be sexually transmitted between humans [1]. *Aedes aegypti* and *Ae. albopictus* inhabit regions with tropical, subtropical, and temperate climates; however, *Ae. albopictus* has a broader range in temperate climates [2]. *Aedes aegypti* is considered the primary vector of ZIKV, dengue virus, and chikungunya virus due to its blood feeding preference for humans, whereas *Ae. albopictus* is an opportunistic blood feeder and will feed on any available host, including humans [2,3]. 

Zika virus is a positive-sense, single-stranded RNA virus of 10,794-nucleotides and has three major lineages: East African, West African, and Asian [4]. Zika virus was first discovered in 1947 in a monkey living in the Zika Forest of Uganda [1,5]. The first documented case of a human being infected with ZIKV was in Nigeria in 1953 [6]. Before the 1980s, ZIKV’s geographic range was restricted to Africa and Asia and the first major human outbreak of ZIKV was on Yap Island, Micronesia (2007) [4,7]. In subsequent years, ZIKV outbreaks were detected in French Polynesia (2013), New Caledonia (2014), Easter Island (2014), the Cook Islands (2014), Mexico (2015) northeastern Brazil (2015), and the first documented case of local transmission of ZIKV in the United States occurred in July 2016 in Florida [4,8,9]. The lineage responsible for the American outbreak of ZIKV is the Asian genotype [3]. Mild symptoms of ZIKV infection include fever, rash, arthritis, and conjunctivitis, while more severe outcomes include neurological (microcephaly in developing fetuses) or auto-immune complications (Guillain-Barre syndrome) [4,9]. The duration of *Ae. albopictus* adult life cycle is 10–46 d (perhaps on the lower end of this range in wild populations), and the extrinsic incubation period (EIP) for ZIKV is approximately 7 d, depending on biological and environmental conditions [4,10,11].

Bifenthrin is a Type 1 pyrethroid that affects the central and peripheral nervous system of invertebrates, such as mosquitoes, by hindering sodium channel gating, leading to death [12]. Insecticides can cause two different types of effects, direct toxic effects (causes mortality) and/or sub-lethal effects. Sub-lethal effects of insecticides can include behavioral (e.g., avoidance) and biological (e.g., changes in fecundity/fertility) changes in surviving insects after coming into contact with a sub-lethal dose of the insecticide [13]. Over time, sub-lethal exposure to insecticides may increase the chance of insecticide resistance.

One of the most concerning consequences of the widespread use of insecticides is the development of insecticide resistance. Several potential pathogen vectors have become resistant to insecticides, hence rendering chemical control ineffective in some regions [14]. Due to recent human epidemics related to ZIKV, expanding ranges of potential vectors, and increasing trends in insecticide resistance of mosquitoes, the need for research on ZIKV is at an all-time high [15,16]. Published information about the vector competence of North American *Ae. albopictus* mosquitoes for ZIKV is currently lacking, despite the ongoing epidemic [10]. Vector competence is the ability for mosquitoes to become infected with and transmit pathogens. It is widely known that not all mosquitoes can become infected with and transmit viruses. Biological and environmental factors may impact mosquito midgut and salivary gland infection and escape barriers for arboviruses and the degree of these effects vary between viruses, mosquito populations, and mosquito species [17]. Vector competence for arboviruses is associated with several anatomical barriers to infection, dissemination, and transmission. These include barriers to midgut infection, midgut escape, salivary gland infection, and salivary gland escape [18]. Vector competence is unique for each virus-vector interaction, and can be diverse, even in populations belonging to a single vector species [19]. Therefore, it is essential to evaluate the competence of mosquitoes for virus infection and subsequent transmission [20]. 

Mosquito age is a biological factor that may influence vector competence. Viruses must undergo an EIP in the potential vector before transmission (via saliva) to a subsequent host can occur [20]. The EIP is impacted by biological and environmental conditions, varies between mosquito-virus systems, and can comprise a significant proportion of the vectors’ lifespan [21]. Another factor that can influence vector competence is the gonotrophic cycle. The duration of the gonotrophic cycle of a vector, and hence the frequency with which it feeds, can influence its vector capacity [22]. Furthermore, the immune response of mosquitoes to infection may weaken with age, hence influencing vector competence [23].

In the absence of a vaccine for ZIKV, the only way to prevent ZIKV infection is to control potential vectors. This can be accomplished by biological control measures, source reduction of oviposition sites, larvicides, and adulticides. Insecticides are widely utilized to control vector populations worldwide, thus reducing the risk of disease [24]. However, mosquitoes are becoming resistant to some insecticide active ingredients [25]. Control programs that extensively use insecticides to suppress potential vector populations without using a surveillance-based targeted approach may promote insecticide resistance [26]. Hence, programs should routinely test for insecticide resistance (e.g., Centers for Disease Control and Prevention [CDC] bottle bioassay) and only target potentially dangerous and/or nuisance adult mosquito populations, when necessary. There is currently a lack of published work evaluating if exposure to sub-lethal doses of insecticides, such as pyrethroids, impact the ability of mosquitoes to become infected with ZIKV [25]. 

The central hypothesis in the current study is that contact with sub-lethal doses of insecticides will increase vector competence, the ability of a mosquito to become infected with and subsequently transmit a pathogen, for ZIKV and that this relationship changes with age. Research aimed at elucidating effects of insecticide-mosquito interactions has largely been focused on vector control and development of resistance. Little attention has been given to the impacts of insecticides on vector competence. Insecticide pressure on mosquito populations is a continuing threat as mosquito control is the primary method of protecting the public from mosquito borne diseases. Consequently, our objectives are to characterize the extent to which mosquito–bifenthrin interactions affect vector competence for ZIKV and determine the extent to which mosquito age at the time of insecticide and virus exposure impacts measures of vector competence for ZIKV.

## 2. Results

### 2.1. Virus Titer of Blood Meals, Blood Feeding Rate, Sample Size and Mortality Rates

The titer of the ZIKV blood meal delivered to all groups was 6.3 logs plaque-forming unit equivalents (PFUeq)/mL. The observed blood feeding rate ranged between 10–20% among the four groups and initial sample sizes (blood fed mosquitoes) were: control (no insecticide) old mosquitoes (n = 29), control young (n = 36), bifenthrin-exposed old (n = 63), bifenthrin-exposed young (n = 50). The mortality rates among the four groups are shown in Table 1. Young mosquitoes were 6–7 d old when blood fed and 13–14 d old at the end of the 7 d EIP. Old mosquitoes were 11–12 d old when blood fed and 18–19 d old at the end of the 7 d EIP. The group experiencing the highest mortality at the end of the 7 d EIP were old mosquitoes exposed to bifenthrin (78%, *p* < 0.01) and the lowest mortality rate was observed in young mosquitoes in the control group (33%). We used ANOVA to compare the mortality rates between all four groups.

### 2.2. Infection Rates, Dissemination Rates, Body Virus Titers, and Leg Virus Titers Between Mosquito Ages

**Figure 1:**
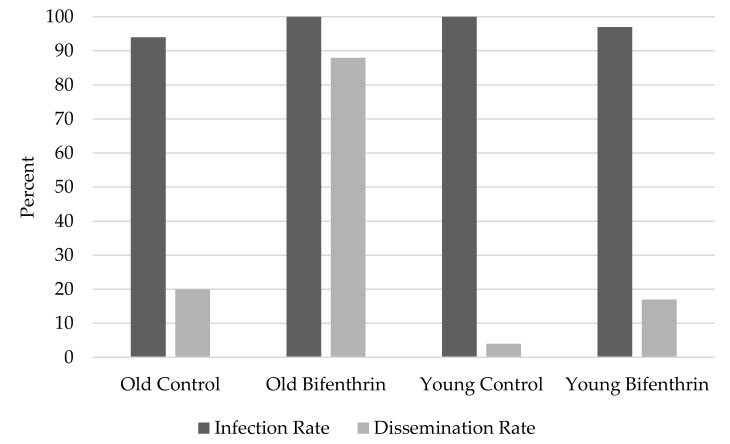
Rates of infection and dissemination in *Aedes albopictus* orally exposed to Zika virus and incubated at 28 °C for 7 d.

No significant differences were observed in infection rates by treatment group (bifenthrin compared to control) in either young (*p* = 0.36) or old (*p* = 0.50) mosquitoes (Figure 1). Dissemination rates were also not significantly different between young (*p* = 0.12) control and bifenthrin-exposed mosquitoes; however, old bifenthrin-exposed mosquitoes showed significantly (*p* < 0.01) higher dissemination rates than old control mosquitoes (Figure 1).

Within the control group, there were no significant differences in infection rate (*p* = 0.39) or dissemination rate (*p* = 0.13) observed between mosquito ages (Figure 1). For mosquitoes exposed to bifenthrin, no significant age differences were observed in infection rate (*p* = 0.65); however, old mosquitoes exposed to bifenthrin exhibited significantly (*p* < 0.01) higher dissemination rates than young mosquitoes exposed to bifenthrin (Figure 1).

**Figure 2:**
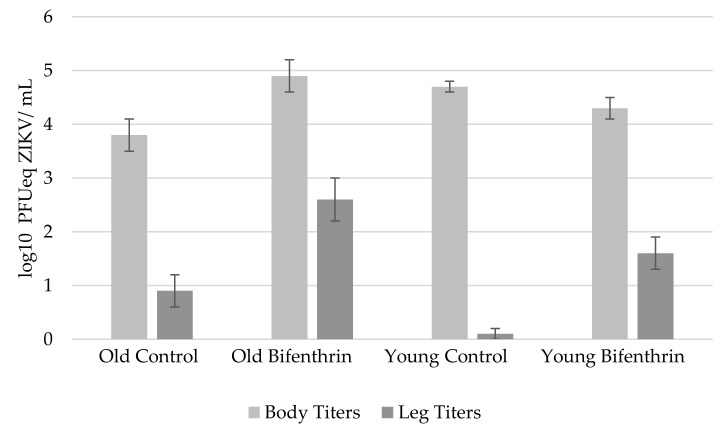
Mean titers ± standard error of *Aedes albopictus* orally exposed to ZIKV and incubated at 28 °C for 7 d.

Young mosquitoes showed no significant differences (*p* = 0.07, *F* = 3.39, df = 1,53) in body titers for those exposed to either bifenthrin or control mosquitoes (Figure 2). However, body titers of old mosquitoes exposed to bifenthrin were significantly (*p* = 0.01, *F* = 7.01, df = 1,30) higher than those of old mosquitoes in the control group (Figure 2). Leg titers were significantly higher in bifenthrin-exposed mosquitoes and this was shown in both young (*p* < 0.01, *F* = 23.76, df = 1,53) and old mosquitoes (*p* < 0.01, *F* = 10.37, df = 1,30) (Figure 2). No significant differences were found in body titers between young and old mosquitoes exposed to bifenthrin; however, significantly higher body titers were observed in young mosquitoes compared to old mosquitoes within the control group (*p* < 0.01, *F* = 9.84, df = 1,39) (Figure 2). Leg titers were significantly higher in old compared to young mosquitoes and this was observed in both the control (*p* < 0.01, *F* = 12.04, df = 1,39) and bifenthrin-exposed (*p* = 0.02, *F* = 6.02, df =1,44) groups (Figure 2). 

## 3. Discussion

We investigated the impact of mosquito age and sub-lethal insecticide exposure on ZIKV infection and dissemination in *Aedes albopictus*. For this population of mosquitoes, mortality rate significantly increased with age. This could be due to older mosquitoes having weakened immune systems that contributed to higher mortality rates after exposed to insecticide stress. This finding is consistent with reported rates of mortality in *Ae. aegypti* where mortality rates were significantly higher in 14 d compared to 3 d old mosquitoes, regardless of whether they were sugar or blood fed [25]. This was also shown in *Anopheles stephensi* where older (10 d old) mosquitoes exposed to 0.25% permethrin-impregnated paper (WHO test kits) showed significantly higher mortality compared to younger (newly emerged) mosquitoes, regardless of resistance or blood feeding status [27]. 

We show that, in old mosquitoes tested here (18–19 d old at the end of the EIP), sublethal bifenthrin exposure significantly increased the ability of ZIKV to disseminate out of the midgut. However, no significant differences were observed between body infection rates as all groups experienced a high degree of infection (≥ 94%). Here, old mosquitoes exposed to bifenthrin exhibited the highest rates of infection and dissemination, as well as the highest leg titers. This could be the result of a lower immune response in older mosquitoes [23]. Here, we used body and leg infection to predict salivary gland infection and transmission. However, not all mosquitoes experiencing body and/or leg infections will be able to transmit ZIKV. Hence, we plan to test mosquito saliva in future studies as a measure of transmission. Low sample sizes (N = 14–30 mosquitoes/group) in the current study should be considered when interpreting results here. Future larger scale studies will examine the extent to which sub-lethal insecticide exposure impacts midgut and salivary gland infection and escape barriers in mosquitoes under a variety of biological and environmental conditions.

Mosquito age is an important factor that can affect vector competence of *Culex quinquefasciatus* for West Nile virus, but the degree of variation may change in different mosquito populations and/or environmental conditions [28]. Here, we show similar results for *Ae. albopictus* and ZIKV. Mosquito age has dynamic properties in the field, hence, vector competence of field populations is variable [28] and this may be difficult to measure in natural populations. This highlights the need for further investigation of the relationship between mosquito age and vector competence. We expect the relationships observed here to change under different biological and environmental conditions, and with different mosquito populations and virus strains. Future studies should explore these factors to determine the extent to which this occurs and if trends can be detected. We show that age and insecticide exposure affect dissemination rates, and this should be considered when developing strategies to reduce vector populations and pathogen transmission. Impacts of insecticides should be considered in risk assessments of potential vector populations. These findings illustrate the importance of evaluating the efficacy of insecticides used in mosquito control programs. By determining the most efficacious product and application rate, the number of mosquitoes surviving the exposure can be reduced. Vector control programs should focus on controlling mosquitoes before adult emergence and/or before mosquitoes have the chance to blood feed and are potentially exposed to pathogens. Younger mosquitoes may have a stronger immune defense against insecticide exposure, hence routine insecticide resistance testing is important.

Insecticides are common stressors for mosquitoes and they are capable of influencing interactions between mosquito vectors and pathogens. Vector competence was significantly affected by age and sub-lethal insecticide exposure in this study. There are several factors that could affect these findings including, but not limited to: mosquito species, mosquito population, mosquito age, virus strain [29], method of exposure of mosquitoes to insecticide, type of insecticide used, and insecticide dose. Consequently, further larger scale studies should be conducted to evaluate a variety of factors that may interact to influence measures of vector competence, including transmission. The implications of this type of study could influence how we look at vector control methods and their role in disease prevention. Therefore, it is vital for us to investigate these types of interactions, so we can better understand the interplay between insecticide exposure and potential disease spread. 

## 4. Materials and Methods 

### 4.1. Mosquitoes and Virus

A colony of *Aedes albopictus* (F_29_) from Louisiana was used in this study. The mosquitoes were reared following standard procedures [28]. Mosquitoes were reared at 28 °C and maintained under a 14 h : 10 h light: dark cycle. Rearing conditions were standardized to generate similar sized individuals. One to two egg strips were placed in each of 11 plastic pans (24 cm × 36 cm × 5 cm) containing approximately 700 mL of tap water. Larvae were fed daily with a 2:1 mixture of liver powder and Brewer’s yeast. Pupae were transferred to 500 mL plastic cups containing approximately 250 mL of tap water. Male and female adults were allowed to emerge and mate in square metal cages (33 cm^3^) with mesh and provided 20% sucrose *ad libitum* [28]. The same procedure was followed with a second set of egg strips (same mosquito colony and generation) being set again five days after the first set. This colony propagation procedure allowed us to use two different chronological ages of mosquitoes (“young” group: 6–7 days and “old” group: 11–12 days post-emergence when exposed to insecticide and fed infectious blood meal). 

### 4.2. Exposure to Insecticide

A standard solution of bifenthrin was prepared by dissolving 12.8 mg of technical-grade active ingredient (Sigma-Aldrich, St. Louis, MO, USA) in acetone (1000 mL) then taking 1 mL of the stock solution and dissolving it into 100 mL of acetone to obtain the 0.128 ug/mL stock solution [30,31]. This bifenthrin dose was used because our previous study determined that this is the amount of bifenthrin residue detected on foliage after a barrier spray was conducted [31]. Bottles were coated with the bifenthrin stock solution (0.128 μg/mL) following the Centers for Disease Control and Prevention (CDC) Bottle Bioassay protocol [32]. Eight 250 ml Wheaton bottles were coated with either 1 mL of bifenthrin stock or 1 mL of acetone (control). The bottles were coated the day before the bioassay and allowed to dry overnight, leaving bifenthrin residue on the internal surface of the bottles. Two groups of female mosquitoes (separated by age) were exposed to the bifenthrin stock in bottles (100 mosquitoes/bottle), young (6–7 d old) and old (11–12 d old) or the control bottles. The bottles were laid on their side and the mosquitoes remained in the bottle for a 30 min exposure period. After exposure, mosquitoes were chilled and transferred to 1 L cardboard cages (returned to incubators at 28 °C) with mesh and provided water. Treatment and control groups were fed an infectious blood meal 24 h post-exposure to bottles. 

### 4.3. Mosquito Infection

Adult female mosquitoes at 6–7 (young) and 11–12 (old) d post-emergence were allowed to feed on cotton pledgets containing a 1:1 mixture of defibrinated bovine blood (Hemostat, Dixon, CA) and ZIKV (Puerto Rico isolate: PRVABC59) supernatant (freshly harvested from Vero cell culture) warmed at 35 °C for 10 min [27,28]. Freshly propagated supernatant was used instead of frozen-thawed supernatant because previous studies have showed diminished rates of mosquito infection using frozen-thawed virus stocks mixed with blood prior to mosquito feeding [33]. Aliquots of 0.1 mL of infected blood were added to 0.5 mL RNA Later and held at −80 °C until processing to determine blood meal titer [28]. Mosquitoes were chilled and fully engorged mosquitoes from each treatment group were transferred to separate 1 L cardboard cages with mesh screening and maintained in incubators for 7 d at 28 °C and provided 20% sucrose *ad libitum* [28].

### 4.4. Blood Meal and Mosquito Processing

Mosquitoes surviving the 7 d incubation period were removed from their cages and killed by holding them in −20 °C freezer. Their legs were removed with forceps. To prevent contamination, forceps were soaked in 70% ethanol and flamed between processing of each mosquito [28]. Each mosquito body and set of legs was placed in separate tubes containing 0.5 mL RNA Later with two 4.5 mm glass beads and stored at −80 °C until further processing. Samples were homogenized at 25 Hz for 3 min (TissueLyser; Qiagen, Valencia, CA, USA) and centrifuged at 4 °C and 3148× *g* for 4 min prior to RNA extraction [25]. 

### 4.5. Virus Assays

Nucleic acids were extracted from each sample using QIAmp viral RNA kit (Qiagen, Valencia, CA, USA) following the manufacturer’s protocol. The amount of viral RNA in each sample was determined using a LightCycler® 480 system (Roche) and Superscript III One-Step quantitative TaqMan reverse transcript-polymerase chain reaction (qRT-PCR) kit (Invitrogen, Carlsbad, CA, USA) [12]. Standard curves used in qRT-PCR were based on 10-fold dilutions of known ZIKV titers determined by plaque assay [30]. 

Virus found in the body but not the legs represented a non-disseminated infection limited to the midgut [30]. Virus found in both the body and legs was considered a disseminated infection [30]. The infection rate was the percentage of mosquitoes with ZIKV-infected bodies. The dissemination rate was the percentage of mosquitoes with ZIKV-infected bodies that also had infected legs.

### 4.6. Statistical Analysis

Viral titers in freshly blood fed mosquitoes as well as body and leg titers at the end of the incubation period were log transformed [log (x + 1)] to improve normality prior to analysis of variance (ANOVA) with the generalized linear model (GLM) procedure in SAS (SAS Institute, Cary, NC). An ANOVA was carried out to examine differences in virus titers between mosquito ages in the insecticide exposure and control groups. Separate ANOVAs were conducted for these two groups (age, treatment/control) to evaluate the individual impact of age and insecticide exposure. Individual mosquitoes were treated as experimental units in these analyses. If significant (*p* < 0.05) differences were observed in an ANOVA, then a Duncan multiple comparison procedure was performed to determine which means were significantly different. 

We conducted two separate analyses of the probability of different body parts (bodies or legs) becoming infected with ZIKV. We analyzed the infection and dissemination rates with respect to mosquito age (young or old) or treatment (bifenthrin or control) using Pearson chi-square tests to evaluate independence in contingency table analyses (*p* < 0.05). We also used Fisher’s Exact Test for the infection and dissemination rates due to the small sample size. 

## Figures and Tables

**Table 1:** Mortality rates of *Aedes albopictus* at the end of a 7 d EIP after feeding on blood infected with ZIKV and incubated at 28 °C.

Group	Sample Size (Day 0)	Sample Size (Day 7)	Mortality Rate (%)
Old Control	29	16	45
Old Bifenthrin	63	14	78
Young Control	36	24	33
Young Bifenthrin	50	30	40
